# Technical Feasibility, Quality and Environmental Impact of a Partial Replacement of Cocoa Nibs with Cocoa Bean Hulls in Chocolate Bars

**DOI:** 10.3390/foods15030558

**Published:** 2026-02-04

**Authors:** Ivana Salvatore, Claudio Beretta, Maria Rudel, Evelyn Kirchsteiger-Meier, Corinna Bolliger, Matthias Stucki, Nadina Müller

**Affiliations:** Department of Life Sciences and Facility Management, Institute of Food and Beverage Innovation, Zurich University of Applied Science (ZHAW), Einsiedlerstrasse 35, 8820 Wädenswil, Switzerland; ivana.salvatore@gmx.ch (I.S.); claudio.beretta@zhaw.ch (C.B.); meryrudel@gmail.com (M.R.); meev@zhaw.ch (E.K.-M.); corinna.bolliger@zhaw.ch (C.B.); matthias.stucki@zhaw.ch (M.S.)

**Keywords:** cocoa bean hulls, product quality, chocolate, environmental impact, novel food status

## Abstract

This study examines the feasibility of incorporating cocoa bean hulls (CBH) into chocolate in order to improve the resource efficiency of the cocoa value chain. The substitution of cocoa nibs with pre-milled cocoa bean hulls without adjustment of fat content was investigated in dark chocolate. The reference R100.0 (dark chocolate, 0% CBH) was compared with V75.25 (25% of cocoa nibs replaced; 16.25% CBH total) and V50.50 (50% replacement; 32% CBH total). Increasing CBH significantly elevated viscosity and yield stress, and firmness rose correspondingly. Both effects align with the literature attributing such increases to higher solids loading and reduced fat content. Colour analysis (ΔE) showed distinct differences between R100.0 and V50.50. Environmental impact was reduced by 16% for V75.25 and 32% for V50.50. According to the EU Novel-Food-Status-Catalogue, CBH is not classified as novel food. While CBH is typically regarded as an underutilized by-product, this study demonstrates its potential as a functional, cost-reducing ingredient in dark chocolate formulations when applied at optimized inclusion levels.

## 1. Introduction

Globally, around 3 billion tonnes of food are lost or wasted each year along the supply chain, contributing substantially to environmental burdens and resource inefficiencies [1,2,3]. In Switzerland, food losses and waste amount to 2.8 million tonnes annually, of which approximately one third occurs during processing [4]. These by-products or side streams from processing often occur in high quantities (e.g., vegetables, fruit, grains and baked goods), have a high environmental impact per amount of product (e.g., meat, cocoa, coffee) or a combination of both.

Cocoa production is associated with disproportionately high environmental and social impacts [5]. In 2023, global cocoa bean production reached roughly 5.5 million tonnes [6], while Switzerland imported and processed about 54,000 tonnes [7]. Given the intensity of land use, deforestation, and carbon emissions linked to cocoa cultivation, the valorization of cocoa processing side streams represents a promising strategy to improve sustainability within the chocolate sector.

One particularly abundant by-product is the cocoa bean hull (CBH), the outer shell which accounts for 10–17% of the cocoa bean mass and is generated during winnowing after roasting [8]. Traditionally, CBH has been discarded, used as animal feed, or directed to low-value applications such as composting or fuel. More recently, interest has shifted toward higher-value applications in food products, owing to its valuable nutritional composition: CBH is characterized by an average protein content of 15% and a total phenolic content of 5.4% [8], alongside a notably high dietary fibre fraction of around 50% [9].

Studies have included cocoa bean hulls in snack products [10], biscuits [11], pork sausages [12], gluten-free bread [13] or in tea formulations [14,15,16] analyzed the influence of CBH in chocolate at different inclusion levels (5–15%). Furthermore, the incorporation of cocoa bean hull fibre has demonstrated potential in previous studies, for example, when applied in muffins [17] or in bread [18]. Chocolate is a rather challenging product, as consumers expect a shiny, smooth surface, optimal texture and melting behaviour as well as a rich flavour profile [19,20,21]. Any change in composition such as the use of cocoa bean hulls potentially induces a change in the rheological behaviour, which then affects the crystallization behaviour and, consequently, the visual appearance, texture and melt of the final chocolate. Several studies have shown the importance of particle size and composition on rheological behaviour and sensory perception [22,23].

While the application of cocoa bean hulls in, e.g., snack products and biscuits, highlights the potential of CBH as a functional ingredient, its use in food products also raises important safety considerations. Cocoa plants can accumulate cadmium (Cd) in the beans, with particularly elevated levels reported in certain growing regions of Latin America [24] where natural Cd levels in the earth’s crust are particularly high. The CBH typically contains higher cadmium concentrations than the nib, often about twice as much, so removing the shell during winnowing is an effective step to reduce Cd in the final product [25]. Beyond heavy metals, other contaminants such as polycyclic aromatic hydrocarbons (PAHs), which may form during roasting or originate from environmental exposure, and mycotoxins like ochratoxin A (OTA) have also been detected in cocoa-derived materials [26]. To mitigate the combined risks of contamination and undesirable sensory effects, the Codex Alimentarius recommends that cocoa mass should not contain more than 5% shell [27]. The shell has often been regarded as contributing little to flavour quality and even as a potential source of off-flavours, and its fibrous texture is thought to complicate grinding and increase equipment wear [28].

Nevertheless, these challenges should not preclude further exploration of CBH as a food ingredient. With careful assessment of safety, processing feasibility and consumer acceptance, the barriers associated with CBH incorporation may be mitigated. If successful, such an approach could not only enhance resource efficiency within cocoa processing but also contribute to more sustainable and innovative chocolate products. To explore this potential, the present study examines the feasibility of CBH incorporation into chocolate and/or cocoa bars, focusing on its effects on processing behaviour, product quality, and safety considerations. If the product is to be marketed, for example, as “chocolate”, the requirements associated with the specific designation must be considered (Ordinance of 16. December 2016 on Foodstuffs of Vegetable Origin, Fungi and Table Salt [29]. For the purpose of this paper, the samples produced will be called chocolate/cocoa bars.

## 2. Materials and Methods

### 2.1. Characterization of Cocoa Bean Hulls

Cocoa bean hulls (CBH) were obtained from Max Felchlin AG (Ibach, Switzerland) in 2024. The CBH is a side product in the couverture production process which originates after the roasting. For the characterization analyses, the CBH were milled using an impact roller mill SR300 (Retsch, Haan, Germany) with a 200 μm sieve.

Fat was determined using a rapid NMR Fat Analyzer ORACLE (CEM Corporation, Matthews, NC, USA). The concentrations of protein, cadmium, acrylamide, aflatoxins, ochratoxin A, aerobic mesophilic bacteria count, yeasts, moulds, *Escherichia coli*, *Bacillus cereus* and *Listeria monocytogenes* were determined by the accredited lab LaborVeritas (Zürich, Switzerland). The concentrations of soluble and insoluble dietary fibres were determined by accredited lab Eurofins (Schönenwerd, Switzerland). All external analyses were done on 1 sample; sampling was done by mixing portions from all the obtained materials to ensure representative results.

With respect to technofunctional quality, the emulsifying stability, foaming capacity and foaming stability, water absorption capacity, water- and oil-holding capacity, pH value and particle size distribution were quantified. These technofunctional analyses described below were performed in triplicate.

#### 2.1.1. Emulsifying Stability (ES) Determination

The ES determination procedure was adapted from [30,31]. Oil-in-water emulsions were prepared in 50 mL Falcon tubes, each containing 15 mL of deionized water (15.0 g) and 15 mL of rapeseed oil (13.8 g; Florin AG, Muttenz, Switzerland). Sample powder was incorporated to achieve a final concentration of 1% (*w*/*w*) relative to the combined mass of water and oil, corresponding to 0.29 g. Emulsions were produced using a polytron homogenizer PT 2500 E equipped with a universal geometry PT-DA 20/2EC-E192 (Kinematica AG, Malters, Switzerland) at 12,462 s^−1^ for 3 min at ambient temperature, while the rapeseed oil was gradually introduced during homogenization. Emulsion stability was assessed at five time intervals: 10 min (t1), 60 min (t2), 3 h (t3), 24 h (t4) and 1 week (t5) after formation. The degree of stability was evaluated by measuring phase separation and comparing the emulsion volume at each time point (tx) to that measured immediately after homogenization (t0). ES was calculated according to Formula (1):

(1) $$ES\;\mathrm{[\%]}=\;\frac{V_{tx}\times 100}{V_{t0}}.$$

$$V_{tx}\;\mathrm{[mL]:}\;Volume\;of\;emulsion\;after\;time\;point\;t_{x}$$

$$V_{t0}\;\mathrm{[mL]:}\;Volume\;of\;emulsion\;after\;emulsification\;t_{0}$$

#### 2.1.2. Foaming Capacity (FC) and Foaming Stability (FS) Determination

The procedure was based on Tsutsui et al. [32], with modifications. FC reflects the proportion of liquid converted into foam immediately after aeration (t0), whereas FS measures foam persistence over time. For testing, a 1:10 (*w*/*w*) ratio of sample to water was used, corresponding to 16.6 g of sample in 150 g of water. The mixture was foamed for 4 min using a milk frother (Lono, WMF, Geislingen an der Steige, Germany). Foam and residual liquid were transferred into a 250 mL measuring cylinder, and the volumes were recorded at t0, 10 min (t1), 60 min (t2) and 3 h (t3). FC and FS were calculated using Formulas (2) and (3):

(2) $$FC\;\mathrm{[\%]}=100-\left(\frac{{V\_Separated}_{t0}}{V_{Total}}\times 100\right).$$

(3) $${FS}_{tx}\;\mathrm{[\%]}=100-\left(\frac{{V\_Separated}_{tx}}{V\_Total}\times 100\right).$$

$$FC\;\mathrm{[\%]:}\;Foaming\;capacity\;after\;foaming\;at\;t_{0}$$

$${FS}_{tx}\;\mathrm{[\%]:}\;Foaming\;stabilty\;at\;time\;point\;t_{x}$$

$${V\_Separated}_{tx}\;\mathrm{[mL]:}\;Unfoamed\;volume\;at\;time\;point\;t_{x}$$

$$V_{Total}\;\mathrm{[mL]:}\;Total\;volume\;before\;foaming$$

#### 2.1.3. Water Absorption (WA) Capacity

The WA assessment followed the method of Lin et al. [33] with modifications. A 4.5 g portion of the sample was mixed with 30 g of water in a 50 mL tube, shaken manually for 1 min, and stored at 4 °C for a minimum of 12 h. The hydrated sample was transferred to a porcelain funnel fitted with a large-pore MN 540 w filter (MACHEREY-NAGEL, Düren, Germany). The retained material was weighed, dried at 40 °C to constant mass, and water uptake was expressed on a dry matter basis using Formula (4):

(4) $$WA\;\mathrm{[\%]}=\frac{\left(m_{wet}-m_{dry}\right)}{m_{dry}}\times 100.$$

$$m_{wet}\;\mathrm{[g]:}\;Mass\;of\;wet\;slurry$$

$$m_{dry}\;\mathrm{[g]:}\;Mass\;of\;dried\;sample$$

#### 2.1.4. Water- and Oil-Holding Capacity (WHC/OHC)

WHC and OHC were determined using a modified protocol from Raikos et al. [34]. In total, 1.0 g of sample and 12 g of either deionized water or rapeseed oil (Florin AG, Muttenz, Switzerland) were combined in a 15 mL centrifuge tube. The mixtures were vortexed for 1 min and centrifuged at 845 rcf for 30 min (Centrifuge 5810, Vaudaux-Eppendorf AG, Schönenbuch, Switzerland). The supernatant was removed, and the residue was weighed to determine retained liquid. WHC and OHC were calculated using Formula (5):

(5) $$WHC/OHC=\;\frac{\left(m_{Water/Oil}-m_{Supernatant}\right)}{m_{Sample}}.$$

$$m_{Water/Oil}\;\mathrm{[g]:}\;Mass\;of\;water\;or\;oil$$

$$m_{Supernatant}\mathrm{[g]:}\;Mass\;of\;supernatant\;after\;centrifugation$$

$$m_{Sample}\;\mathrm{[g]:}\;Mass\;of\;sample$$

#### 2.1.5. pH Determination

A 1:10 (*w*/*w*) dilution of the sample with distilled water was prepared and thoroughly mixed. pH was measured using a calibrated pH meter (CH 837, Schott, Mainz, Germany).

#### 2.1.6. Particle Size Distribution

Particle size was analyzed using a Camsizer X2 equipped with the X-Jet applicator (Microtrac Retsch GmbH, Haan, Germany) under 3 bar compressed air. Measurements were initiated at 70% nominal area density, with images captured by CCD-Basic and CCD-Zoom cameras. Each run concluded after capturing 40,000 images or when low-density images reached 0.01%. Volumetric size distribution data were processed using the Camsizer X2 software (version 6.10.4.1221).

### 2.2. Chocolate and Cocoa Bar Production

**Figure 1:**
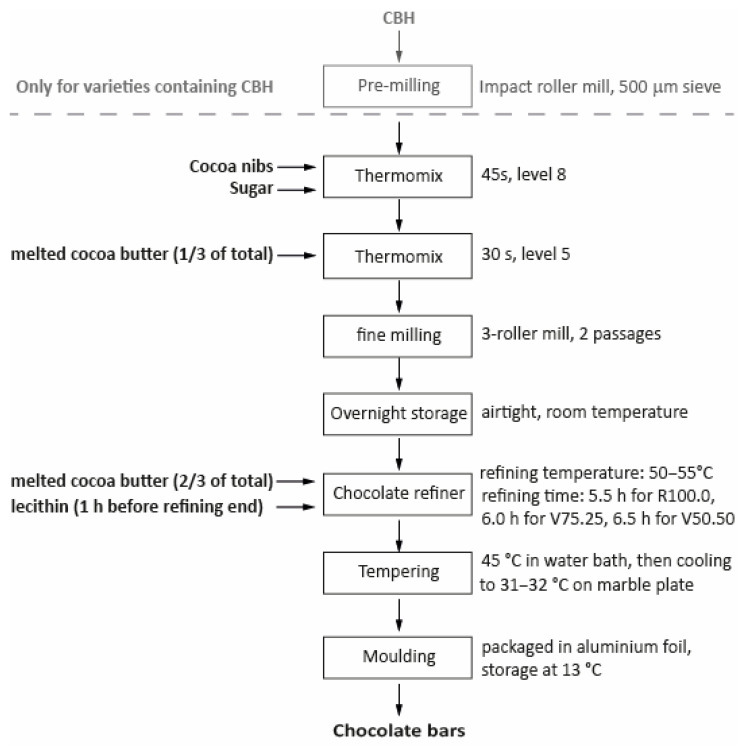
Flow chart of the chocolate production process for the reference chocolate R100.0 and the two varieties with CBH V75.25 and V50.50. The steps above the dashed line were only done for chocolate varieties with cocoa bean hulls.

The production process is shown as a flow chart in Figure 1 where the first step is only valid for chocolate containing CBH, while the rest of the process was identical for all chocolate bar varieties.

Cocoa nibs (origin Ghana, Barry Callebaut, Zürich, Switzerland) and sugar (fine granulation, Ø 0.20–0.90 mm, Schweizer Zucker AG, Frauenfeld, Switzerland) were pre-mixed in a Thermomix (TM31 Vorwerk, Wuppertal, Germany) for 45 s on level 8. If the recipe contained CBH, it was pre-milled using an impact roller mill SR300 (Retsch, Nordrhein-Westfalen, Germany) with a 500 μm sieve and pre-mixed in the Thermomix together with the cocoa nibs and sugar. For each formulation, a third of the total cocoa butter (origin Dominican Republic, Max Felchlin AG, Ibach, Switzerland) was melted in a water bath and added before mixing for 30 s on level 5. Subsequently, the obtained mixtures were transferred to a three-roller mill (SDY 200, Bühler, Uzwil, Switzerland) and milled in two consecutive stages. During stage 1, the mechanical gaps were set to 0.207 mm (feed-centre) and 0.138 mm (centre-apron). The nip load applied via the hydraulic system was set to 8 bar. In the second stage, the gaps were reduced to 0.069 mm and 0.023 mm, and the hydraulic loading pressure was increased to 16 bar. This procedure resulted in pre-milled powder-like mixtures, which were stored in airtight conditions and at ambient temperature overnight. The following morning, the mixtures were processed in a chocolate refiner (PG 508, Premier, Chennai, India) whilst heating the refiner wall with a heat gun (HL 1920 E, Steinel-Tools, Herzebrock-Clarholz, Germany) at setting 2, leading to a product temperature of approximately 50 °C, but not exceeding 55 °C.

Because the CBH-containing formulations exhibited reduced overall fat content and higher viscosity, a portion of the remaining two thirds of cocoa butter was added earlier than for the reference products to ensure stable refining. The total cocoa butter amount per formulation remained identical. After 3 h of refining, the rest of the melted cocoa butter remaining for each formulation was added. The particle size of the mixtures was monitored using a digimatic micrometer (MDE-25PX, Mitutoyo, Neuss, Germany) by performing a triplicate measurement approximately every 60 min. 1 h before ending the refining process, rapeseed lecithin was added to the mixtures. The total refining times differed for each variant, primarily reflecting the time required to reach the target particle size. It was 5.5 h for R100.0, 6 h for V75.25 and 6.5 h for V50.50. Tempering was done by heating the cocoa mass to 45 °C in a water bath, pouring one third on a marble plate and cooling it to 24–26 °C. The cooled mass was then mixed with the remaining mass and the procedure was repeated until the temperature reached 31–32 °C. The mass was poured into curing moulds and stored at 13 °C overnight. It was then wrapped in aluminum foil and kept at 13 °C until further use.

Three chocolate/cocoa bar formulations were produced on two different trial days each. The compositions of the formulations are listed in Table 1. R100.0 refers to the reference formulation, a dark chocolate not containing any CBH. In the V75.25 variant, 25% of the cocoa nibs were substituted through CBH, resulting in 16.25% CBH in the formulation. Similarly, V50.50 replaces 50% of the cocoa nibs from the original formulation through CBH, resulting in 32.5% CBH in the total formulation. Per variant 600 g was produced per trial.

### 2.3. Chocolate Analysis

The different chocolate varieties were analyzed with respect to their colour and texture in solid state and the viscosity in molten state. All analyses were performed 1 week after production to ensure comparable sample ageing.

#### 2.3.1. Colour

The analysis of the L*a*b* spectrum was performed on all bars produced once and, in total, 6 times on the top surface and 6 times on the bottom surface of the samples using the instrument Chromameter CR400 (Konica Minolta, Chiyoda, Japan). In the Lab* colour space, L* represents lightness, a* indicates the red–green axis and b* corresponds to the blue–yellow axis. Colour difference (ΔE) was calculated using Formula (6) and used to quantify visually perceptible changes. These differences are categorized as very distinct (ΔE > 3), distinct (1.5 < ΔE < 3), or slight (ΔE < 1.5) [35].

(6) $$\Delta E=\sqrt{\left(\Delta L^{*2}+\Delta a^{*2}+\Delta b^{*2}\right)}$$

In addition to the chocolate/cocoa bar analyses, the colour of the unmilled CBH and cocoa nibs was measured in triplicate in identical fashion.

#### 2.3.2. Firmness

Firmness measurement of the chocolate/cocoa bars was adapted from Afoakwa et al. [36] and was measured using a texture analyzer (Model TA-XT Plus, Stable Micro Systems, Godalming, UK) equipped with a 50 kg load cell and a 2 mm needle probe. Samples (5 mm × 36 mm × 22 mm) were conditioned to the room temperature of the air-conditioned laboratory. Test parameters were set as follows: pretest speed of 1.0 mm/s, test speed of 2.0 mm/s, post-test speed of 10.0 mm/s, trigger force of 20 g, and a penetration distance of 4 mm. Twelve replicate measurements on two bars were performed per production trial. The peak force was recorded as the firmness, and the work of penetration was calculated as the area under the force–time curve up to the peak force.

#### 2.3.3. Rheology

The rheological analysis of the chocolate masses was performed according to the official Method 46 of the International Office of Cocoa, Chocolate and Sugar Confectionery (‘Viscosity of Cocoa and Chocolate Products’) [37]. The cylinder had a length of 39.999 mm and a diameter of 26.659 mm, while the cup had a diameter of 28.934 mm, resulting in a gap of 1.1375 mm (CC27/T200/AL, Anton Paar GmbH, Graz, Austria). The analysis was performed on an Anton Paar Rheometer (Dynamic Mechanical Analyzer, MCR 702, Graz, Austria) and in triplicate per trial, resulting in six total measurements. After temperature equilibration at 40 °C for 180 s (±0.2 °C), samples underwent pre-shearing at 5 s^−1^ for 180 s. Subsequently, a shear rate ramp was applied from 1 to 50 s^−1^ over 180 s; then, the shear rate decreased from 50 back to 1 s^−1^, including a 60 s hold at 50 s^−1^. Data acquisition and evaluation were carried out using RheoCompass 1.25.373 by Anton Paar. Flow curves were fitted with the Windhab model, from which yield stress and apparent viscosity were derived [38].

#### 2.3.4. Storage Test

To determine the effect of storage on the rancidity of the chocolate/cocoa bars, samples were sent in for analysis one day after production and after three months of storage at 13 °C. Hexanal and free fatty acids (FFA) were determined by the accredited lab UFAG (Sursee, Switzerland).

#### 2.3.5. Final Composition

The nutritional profiles of the chocolate/cocoa bars, as well as critical components, such as cadmium and acrylamide, were calculated using the manufacturers’ specifications for the individual ingredients. Since no specifications were available for CBH, the measurement data of moisture, fat, protein, soluble and insoluble fibres, acrylamide and cadmium were used for the calculations. Carbohydrate content was calculated by difference using the measured components.

### 2.4. Environmental Assessment

In this study, a life cycle assessment (LCA) was conducted to evaluate the environmental performance of valorization and utilization scenarios for cocoa bean hulls. The LCA followed the four steps described by ISO 14040 and 14044 standards [39,40] and was performed using the SimaPro software 10.2 (PRé Sustainability, Amersfoort, The Netherlands). The impact assessment was carried out using the comprehensive ecological scarcity method that covers 19 environmental impact categories [41]. Each impact is characterized by an eco-factor based on the distance-to-target principle, which compares current emission flows to target limits. Results for the single-score indicator are expressed in eco-points (EP), allowing for direct comparison across products [41]. Additionally, the life cycle greenhouse gas emissions using the IPCC 2021 GWP 100 method [42] was computed for the different chocolate/cocoa bar scenarios. Modelling was based on primary data from this study and background data from ecoinvent v3.11 [43]. The assessment covered the full life cycle from raw material extraction to disposal (i.e., cradle to grave).

In a first assessment, the environmental impacts of integrating CBH into dark chocolate was quantified for a functional unit of 1 kg of final product ready for use. Although CBH are a by-product, they hold economic value. Accordingly, environmental impacts were distributed between the main products and side streams using economic allocation reflecting relative market values. For CBH, 1.1% of the total value is attributed to the side stream, while the remaining 98.9% is assigned to the cocoa bean (see Supplementary Material).

In a second assessment, valorization pathways with alternative utilization scenarios (feed, heat and electricity co-generation via incineration, and mulching) were compared. For this, a system expansion with substitution approach was applied. The functional unit for the comparison was defined as the valorization or utilization of 1 kg of CBH, enabling consistent comparison across heterogeneous outputs. Each output was related to the quantity of a reference product substituted when side stream-based alternatives enter the market. Substitution products included reference dark chocolate (R100.0, produced solely from cocoa beans) for the two side stream-based dark chocolates (V75.25 and V50.50). For mulching, substitution was based on the equivalent NPK content of mineral fertilizer. For incineration, the substituted products were the Swiss average electricity mix and natural gas (central or small-scale) for the energy produced. Feed substitution was modelled with a mixture of wheat bran, oat husks, sunflower cake, and alfalfa cubes providing the same nutrients. Details on system boundaries, reference products, inventory data (Tables S1–S9) and allocation procedures (Table S10, and Figure S1) are provided in the Supplementary Material [44,45,46,47,48,49,50,51,52,53,54].

### 2.5. Establishment of Novel Food Status

For the regulatory analysis to establish the novel food status of CBH, the following sources were consulted and evaluated:

- Commission Implementing Regulation (EU) 2017/2470 of 20 December 2017 establishing the Union list of novel foods in accordance with Regulation (EU) 2015/2283 of the European Parliament and of the Council on novel foods (Commission Implementing Regulation (EU) 2017/2470 of 20 December 2017 establishing the Union list of novel foods in accordance with Regulation (EU) 2015/2283 of the European Parliament and of the Council on novel foods, OJ L 351, 30.12.2017, p. 72–201, last consolidated version: 20 August 2025) [55].
- Federal Food Safety and Veterinary Office (FSVO). Website: Authorization of Novel Foods [56].
- European Commission. Website: Consultation process on novel food status [57].
- European Commission. Website: EU Novel Food Status Catalogue [58].

### 2.6. Statistical Testing

Statistical analyses were performed in RStudio (Version 2023.03.0, Posit PBC, Boston, MA, USA). Data are reported as mean ± standard deviation (SD). Group differences among variants were tested with Kruskal–Wallis (α = 0.05) and, when significant, pairwise Wilcoxon rank-sum tests with Holm adjustment were applied. For colour (L*, a*, b*), a two-way MANOVA (Variant × Side) using Pillai’s trace assessed overall effects, followed by univariate nonparametric comparisons as above.

## 3. Results and Discussion

### 3.1. Characterization of Cocoa Bean Hulls

In Table 2, the milled cocoa bean hulls are characterized for beneficial and critical components and properties. This includes the nutritional composition, potential biological and chemical contaminants and the results of the technofunctional assessment.

The findings are a point-in-time assessment. Microbiological and chemical risk profiles can vary with raw material origin and season, storage, and process parameters; thus, the absence of hazards in the present lots does not guarantee their absence in future batches. In practice, verification should be maintained within the HACCP framework, particularly whenever raw materials or processing conditions are changed.

The measured moisture content of 5.9% falls within the range of 4.7–10.2% reported in the literature for various types of CBH [9,59,60,61,62]. The fat content was determined to be 6.4%, which falls within the range of 6.62–18.5% reported in the literature [9,59,60,61,62]. CBH contain substantially less fat than cocoa beans, which have an approximate fat content of 50% [63]. As a consequence, when incorporating CBH into food formulations, an adaptation of the fat content could be considered in order to limit a change in flow behaviour. Both the protein content (15.5%) and the total fibre content (54.6%) were within the literature ranges of 11.6–20.9% for protein and 50.4–60.6% for fibre [9,59,60,61,62]. Nibs show a moisture content of 1.6–6.6% [64,65,66] after drying, protein about 12.5–16.7% [67,68], and markedly lower total dietary fibre 12.8–16% [65,69] than CBH. Relative to nibs, replacing a fraction with CBH will therefore leave protein in a comparable range but substantially elevate dietary fibre, which may warrant adjustments to the process or formulation to maintain target flow behaviour.

Cadmium is a toxic heavy metal that can be absorbed by cacao trees from certain soils and thus contaminate cocoa products. Consequently, the Swiss Ordinance on the Maximum Levels for Contaminants (ContO) (Ordinance of 16 December 2016 on the Maximum Levels for Contaminants) [70] sets concentration limits for cadmium in cocoa powder and chocolate products. For cocoa powder, the maximum allowed concentration is 0.6 mg/kg, and for chocolate with a cocoa content of ≥70%, 0.9 mg/kg. Although CBH are not yet specifically regulated by the Swiss law, they are known to sometimes contain an increased cadmium content due to their high absorption capacity, with values reported from 0.124 mg/kg [71] to 0.629 mg/kg [72]. As a result, 0.37 mg/kg measured in the CBH falls within a typical range and is factor 2.4 below the maximally allowed concentration for chocolate with a cocoa content of ≥70%. Based on the sample V75:25 where 25% of the cacao nibs was replaced by CBH, 16.25 g of a 100 g chocolate bar are CBH and the total cadmium content is 0.0060 mg per 100 g bar chocolate. The European Food Safety Authority EFSA limits the tolerable weekly intake (TWI) for Cd to 2.5 micrograms per kg of body weight [73]. Hence, a child of 20 kg body weight should consume no more than 0.119 kg V75:25 chocolate per day.

According to the ContO, the maximum content for ochratoxin A in cocoa powder is 3 µg/kg (Ordinance of 16 December 2016 on the Maximum Levels for Contaminants) [70], which can serve as a reference value for CBH, as no specific limit has been established for CBH and cocoa powder is the most comparable raw material. Studies have shown that ochratoxin A levels in roasted CBH (6.53 µg/kg) can be approximately five times higher than in roasted cocoa nibs (1.36 µg/kg) [74], underscoring the higher contamination risk that must be taken into account when incorporating CBH into food products. In the present study, the ochratoxin A concentration in CBH measured was 0.37 µg/kg, which is relatively low and does not indicate a substantial risk; however, the potential for contamination should still be carefully assessed whenever incorporating CBH in food products. No aflatoxins were detected in the present samples. Previous studies, however, have reported their occurrence in CBH, where they tend to appear more frequently than in other parts of the bean [75]. The deviations in mycotoxin contents are with high probability caused by different weather conditions before and during harvest or differing storage conditions [74].

An acrylamide content of 273 μg/kg was found in the present CBH. This value falls in the upper range reported by Żyżelewicz et al. [76] for cocoa beans (62.33–275.39 µg/kg) and is comparable to values found by Granvogl and Schieberle [77] in cocoa masses (63 to 643 µg/kg). Currently, no legislative guidelines exist for cocoa or products thereof. However, for similar products as biscuits, cookies, etc., a value of 300 μg/kg is specified (Ordinance of 16 December 2016 on the Maximum Levels for Contaminants [70]. Hence, the acrylamide content in the CBH is below but close to the legislative guideline for biscuits and cookies.

Aerobic mesophilic germs, yeasts, mould, Escherichia coli, Bacillus cereus (presumptive), and Listeria monocytogenes were all not detectable in the present CBH.

The WHC was determined to be 357.2% and the OHC 238.1%. The WA was measured at 261.4%. Rinaldi et al. [13] reported WHC values of 308–350% for the CBH of three different particle size fractions, which are in good agreement with the present findings. For WA, Rinaldi et al. [13] reported 398–552%, which is notably higher than in the present study; however, methodological differences between the studies limit the direct comparability of these results. High WA and WHC values can play an important role during storage of the finished product as chocolate is a low moisture product and the absorption of water could affect quality. An increased water content can act as an accelerant of enzymatic oxidation [78] and, furthermore, lead to a moistening of sugar crystals, thus promoting sugar migration to the chocolate surface where it leads to sugar bloom (visual and textural impairment) [79].

Compared with roasted cocoa nib powder, exhibiting a WHC of 134–202% [80], the higher WHC value of CBH (357.2%) is consistent with the nibs’ higher fat and lower fibre matrix. Their OHC is 129–163% [80], again below the measured CBH OHC (238.1%). A higher OHC implies that more fat is bound within the CBH than within a standard chocolate matrix which can lead to a change in viscosity [81] affecting smoothness during melting of the chocolate in the mouth. Furthermore, Aidoo et al. [82,83] showed that the addition of fibres can increase or decrease the firmness of the final chocolate depending on the fibre properties such as particle morphology and solubility.

Goude et al. [84] reported foam capacity for cocoa flour at 8.6% (±2.1%), comparably low compared to the CBH capacity of 6.4% (±1.0%). Cocoa particles have shown potential as Pickering stabilizers for O/W emulsions, although their effectiveness is heavily dependent on particle fineness. Hence, no change in technofunctionality with respect to foaming behaviour is expected for CBH-enriched chocolate.

### 3.2. Chocolate Characterization

Based on the evaluation of the nutritional quality, contaminant levels and technofunctional behaviour of CBH, the application in chocolate as a partial replacement of cocoa nibs seemed feasible. In order to achieve a sustainability benefit, high amounts of cocoa nibs were replaced (25 and 50%) and the resulting chocolate analyzed.

#### 3.2.1. Particle Size During Production

**Figure 2:**
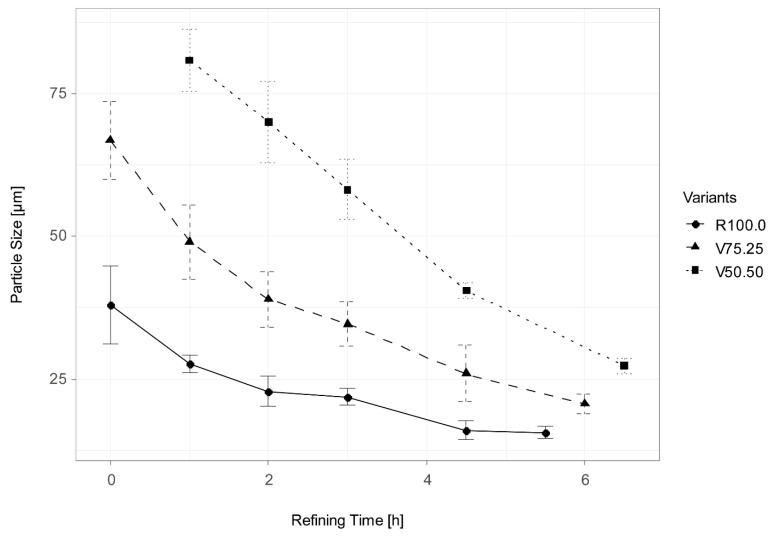
Particle size over refining time. R100.0 = reference sample, V75.25 = sample with cocoa bean hulls substituting 25% of cocoa nibs, V50.50 = sample with cocoa bean hulls substituting 50% of cocoa nibs.

During production, higher CBH incorporation led to larger particle sizes in the chocolate masses. Figure 2 shows the measured particle size over refining time. After refining, the mean particle size (±SD) was 15.7 ± 1.0 µm for the reference (R100.0), 20.7 ± 1.8 µm for V75.25, and 27.3 ± 1.4 µm for V50.50. For dark chocolate, particle size strongly influences mouthfeel: particles above ~30 µm tend to feel gritty, while particles ≤ 25 µm are typically below oral detection thresholds [85]). In this context, the mean sizes observed here, 15.7 µm (R100.0), 20.7 µm (V75.25), and 27.3 µm (V50.50), place the reference within the “smooth” range and the CBH variants near, but generally within, widely accepted limits for dark chocolate texture.

#### 3.2.2. Colour

**Figure 3:**
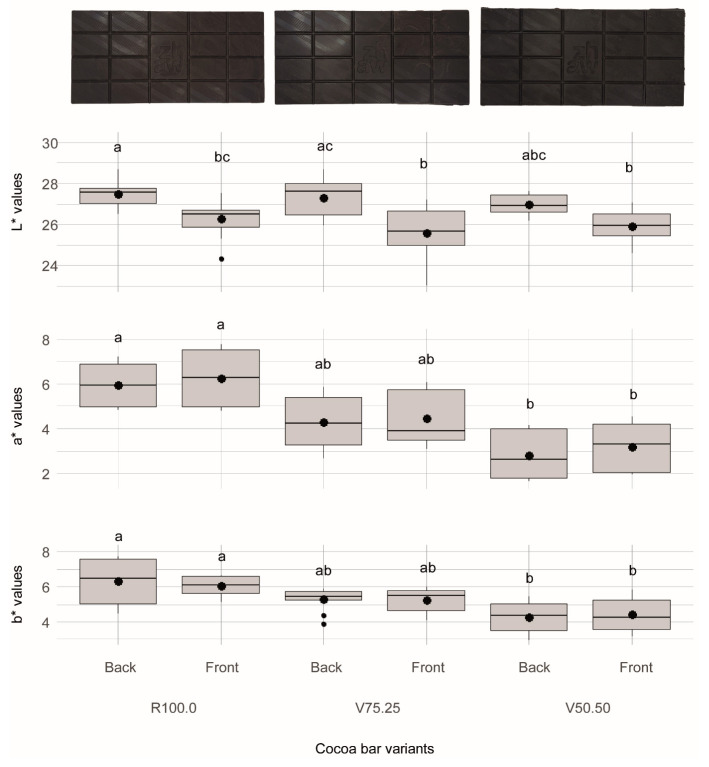
L*a*b* values of chocolate/cocoa bars measured from the front and the back. R100.0 = reference sample, V75.25 = sample with cocoa bean hulls substituting 25% of cocoa nibs, V50.50 = sample with cocoa bean hulls substituting 50% of cocoa nibs. Compact letters (a, b, c) indicate groups of significance, i.e., group means not sharing any letter are significantly different.

The colour analysis revealed significant differences in lightness between the measurements done from the front and from the back of the chocolate/cocoa bars (see Figure 3). One reason could be a more uneven structure of the back side caused by the subseparation of differently sized particles. A tendency of a decrease in a* and b* with increasing addition of cocoa hull as well as a significant difference between R100.0 and V50.50 was observable. Overall, the present results indicate that the more CBH is integrated, the lower the red and yellow tones and the more it moves more towards green and blue tones. However, no clear trends nor significant differences were observable for the L* values. Conversely, Barišić et al. [16] reported darker chocolates with CBH inclusion, which could relate to fibre particle size, shell treatment, or the two-month storage window examined.

The raw material colour measurements yielded the following means: CBH L* 41.18 (±1.02), a* 4.13 (±0.42), b* 4.77 (±0.61); cocoa nibs L* 43.18 (±1.15), a* 5.51 (±0.27), b* 5.66 (±0.38). The lower a* and b* values of the CBH relative to the nibs likely contributed to the decreases in a* and b* observed in the bars containing CBH.

Öztürk and Ova integrated milled CBH in pound cakes and observed that L*, a* and b* values in crust and crumb decreased linearly to CBH inclusion [86]. Similarly, substituting wheat flour with CBH in chocolate cakes caused only the a* and b* value to significantly decrease [87], aligning with the present results. In contrast, another study included CBH in gluten-free bread and observed a significant reduction in L* and simultaneously an increase in a* and b* values in the crumb [13]. The difference in findings in this study and that of Öztürk and Ova is in the baking process where a change in composition can affect the extent of browning of the product.

Colour differences (ΔE) were small between the front and back surfaces of the same variants. Across variants, V75.25 differed distinctly from both R100.0 and V50.50 (ΔE = 1.56–2.62). The largest shifts were observed for V50.50 vs. R100.0, which showed very distinct differences (ΔE = 3.48–3.92). The complete pairwise ΔE matrix is provided in the Supplementary Information.

#### 3.2.3. Firmness

**Figure 4:**
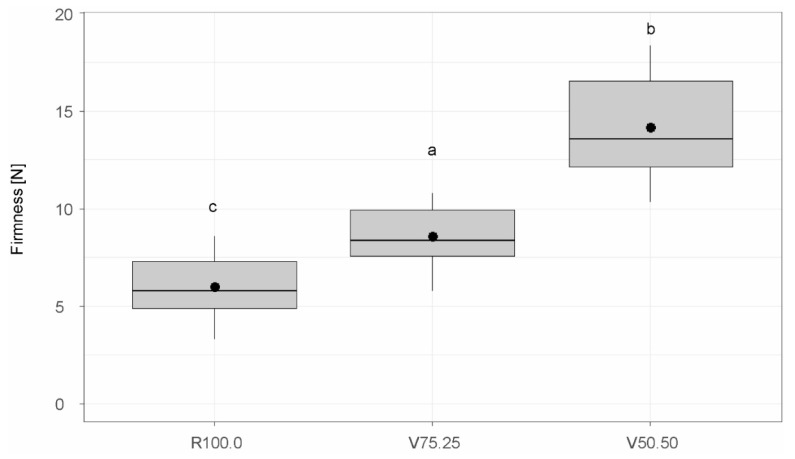
Firmness values of chocolate bars. R100.0 = reference sample, V75.25 = sample with cocoa bean hulls substituting 25% of cocoa nibs, V50.50 = sample with cocoa bean hulls substituting 50% of cocoa nibs. Compact letters (a, b, c) indicate groups of significance, i.e., group means not sharing any letter are significantly different.

A distinct and significant increase in firmness with increased CBH content was observable, as seen in Figure 4. Specifically, while R100.0 exhibited a firmness of 6.02 ± 1.60 N, the values increased significantly to 8.60 ± 1.56 and 14.2 ± 2.63 with higher CBH incorporation.

The firmness of dark chocolate is influenced by compositional and structural factors, including particle size distribution (PSD), dietary fibre and fat content and emulsifier level. Previous studies have demonstrated that finer particles produce a denser sugar crystalline network and stronger inter-particle interactions, resulting in higher firmness values [88]. In the present study, however, the variant with the largest average particle size (V50.50) exhibited the highest firmness. This discrepancy may be explained by the stronger influence of other factors, particularly the reduction in fat content and the increasing fibre fraction upon the introduction of cocoa bean hulls, which are known to exert greater effects than PSD on rheological and textural properties [36,89].

Afoakwa et al. [88] found that chocolates formulated with lower fat levels exhibited higher firmness. In the current samples, reducing fat content from 50.4% (R100.0) to 42.6% (V75.25) and further to 34.7% (V50.50) corresponded with a steady increase in firmness, confirming this relationship.

Additionally, fibre type and shape modulate chocolate hardness. Studies on soluble fibre in sugar-free dark chocolates report mixed effects: depending on solubility, particle morphology, water activity, and tempering, this fibre can either increase or decrease hardness [82,83]. However, the use of CBH introduces more insoluble fibres (39.9%) than soluble fibres (14.7%). In our samples, firmness increased with higher CBH, consistent with dilution of the effective fat phase and higher solids load. Because many types of dietary fibre are hygroscopic, fibre-enriched chocolates are more prone to moisture uptake. A study by Verde et al. [90] found that fibre addition in chocolate is associated with greater moisture uptake and comparatively lower hardness over storage, relative to the control chocolate. As moisture and water activity were not measured here, a moisture contribution cannot be excluded.

Finally, it should be noted that lecithin concentrations, tempering and storage conditions also affect firmness and texture. However, since these parameters were held constant across all variants, they are unlikely to account for the differences observed.

#### 3.2.4. Rheology

**Figure 5:**
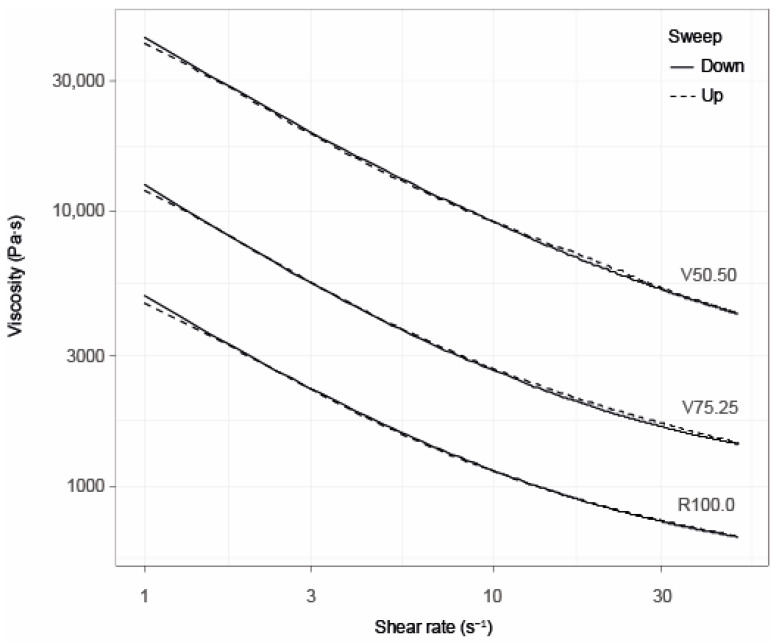
Viscosity of chocolates at different shear rates. R100.0 = reference sample, V75.25 = sample with cocoa bean hulls substituting 25% of cocoa nibs, V50.50 = sample with cocoa bean hulls substituting 50% of cocoa nibs.

Figure 5 shows the viscosity over the shear rates measured and Table 3 lists the calculated Windhab parameters. As with firmness, the viscosity and flow behaviour of melted dark chocolate seem to be mainly determined by PSD, fat content and emulsifiers [91]. Consistent with earlier studies, lower fat contents upon increasing additions of CBH significantly increased viscosity across all shear rates due to enhanced particle–particle interactions caused by a reduced lubrication effect at lower fat content [36]. Across the literature, results suggest that adding other fibre- or polyphenol-rich ingredients to chocolate can also raise viscosity [89]. In line with the Krieger–Dougherty equation, lowering fat content raises viscosity unless other aspects of the formulation and production protocol are adjusted [92]. Deou et al. [93] show that adjusting the PSD can enhance packing density sufficiently to keep viscosity constant at lower fat levels. Furthermore, Feichtinger et al. [94] confirmed that PSD and size ratio strongly determine viscosity in chocolate-like systems. In our case, fat decreased and mean particle size increased without PSD and packing density optimization, resulting in increased viscosity of the mass.

The findings are supported by the parameters of the Windhab model (see Table 3) for the different flow curves where both the basic yield stress, i.e., the transition from solid-like to liquid-like, and the higher limiting yield stress are higher at 25% CBH than at 0% and highest at 50% CBH. In Windhab terms, a higher τ_0_ denotes a stronger “state of rest structure”, a higher τ_1_ indicates more pronounced shear-induced structuring, and a higher η∞ (high-shear slope) reflects greater resistance at high shear. Collectively, these results indicate a stepwise strengthening of the rest structure (τ_0_), enhanced shear-induced structuring (τ_1_) and increased high-shear resistance (η∞) with rising CBH. The rise in D* only at 50% CBH suggests a threshold-like increase in stress sensitivity. These rheological shifts are consistent with a reduced free-fat phase and higher solids/fibre loading at elevated CBH levels.

Emulsifiers such as lecithin and polyglycerol polyricinoleate (PGPR, E476) lower viscosity and yield stress by reducing particle friction and improving wetting. Their effects are most pronounced at low fat levels. Despite decreasing fat levels, maintaining lecithin at a constant 0.5% yielded no observable reduction in viscosity. However, adjusting the type or ratio of emulsifiers may help offset increases when using fat-replacing ingredients such as CBH [36,95,96].

Moisture also modulates the flow properties of chocolate; even slight increases can cause sugar agglomeration and increase viscosity. When hygroscopic fibres are incorporated, moisture uptake may be enhanced, further amplifying this thickening effect [89].

Overall, the present results indicate that fat is the dominant factor controlling viscosity, while PSD, emulsifiers and fibres act as secondary but complementary modifiers.

#### 3.2.5. Storage Test

Table 4 shows the measured FFA and hexanal values. Before storage, the reference R100.0 showed an FFA content of 1.1 g/100 g and hexanal content of 0.3 mg/kg. When including CBH, the FFA content before storage in V75.25 (1.2 g/100 g) and V50.50 (1.7 g/100 g) were slightly increased. Hexanal values before storage were all similar, with levels of 0.2 mg/kg in V75.25 and 0.3 mg/kg in V50.50. Sensorial rejection associated with free fatty acids is highly dependent on the specific fatty acid involved. Running et al. [97] found no rejection up to concentrations of 2.6% free fatty acids from stearic acid, an aroma threshold upon the addition of 1.76% oleic acid and no taste threshold up to 2.5%, while for linoleic acid, the threshold for aroma was low with 0.36% and for taste was found to be 2.2%. Based on these findings, it can be deduced that the FFA values in both the reference samples and samples with CBH are with high probability below a sensorially detectable level.

After 3 months of storage, only minor changes in FFA and hexanal levels were observable. While CBH is a source of antioxidants [98], their potential positive effect was not observable in this period of time and would need further investigation over a longer storage period.

#### 3.2.6. Final Composition

Table 5 presents the nutritional values of the chocolate samples calculated. The most notable change is the reduction in fat content as cocoa nibs are increasingly replaced with CBH, given that cocoa nibs contain approximately 50% fat versus 6.4% for cocoa bean hulls. This fat reduction leads to a corresponding decrease in total calorie content. Additionally, dietary fibre content increases strongly from 0 g/100 g to 9 g/100 g and 18 g/100 g, allowing the V75.25 and V50.50 variants to qualify as “high-fibre” products according to the Ordinance on the Information on Foodstuffs (FoodIO) (Ordinance of 16 December 2016 on Information on Foodstuffs [99]. Although there is a slight rise in protein content, it remains insufficient to support any further nutritional claims.

An internally conducted screening regarding the sensorial properties of the final chocolate/cocoa bars revealed an increased stickiness and astringency with increasing CBH content. The intensity of individual aroma attributes was slightly modified by the addition of CBH, but no off-flavours were observed. The results are an initial indication of the influence of the modification on the sensory properties of the chocolates.

### 3.3. Environmental Impact

**Figure 6:**
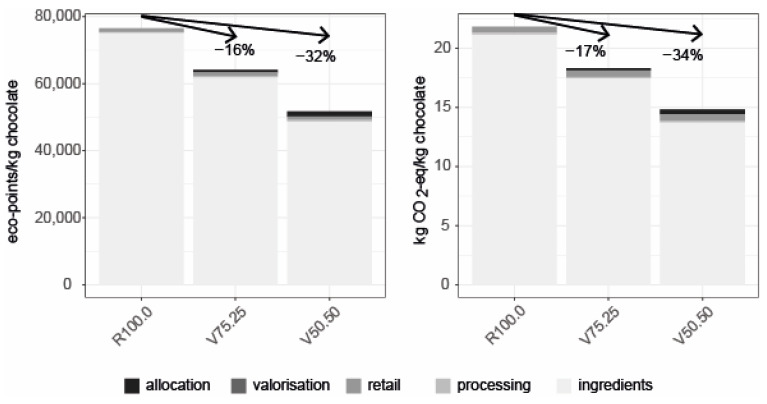
Overall environmental impacts (eco-points, left) and life cycle greenhouse gas emissions (kg CO_2_-eq, right) of chocolate produced with partial substitution of cocoa nibs by CBH, assessed using the ecological scarcity method and the IPCC 2021 methodology, respectively. Retail (packaging, sale), processing, and ingredients are all part of the chocolate production. R100.0 = reference sample, V75.25 = sample with cocoa bean hulls substituting 25% of cocoa nibs, V50.50 = sample with cocoa bean hulls substituting 50% of cocoa nibs.

In this study, CBH were incorporated into dark chocolate production as a partial substitute for cocoa nibs, thereby reducing the environmental impacts per kilogram of chocolate (Figure 6). Substituting 25% of cocoa nibs with CBH (V75.25) reduces the impact by 16.0% compared to the reference chocolate formulation R100.0, while a 50% substitution (V50.50) yields a 32.0% reduction. Comparable reductions are observed for greenhouse gas emissions (GHG): R100.0 amounts to 21.8 kg CO_2_-eq/kg chocolate, V75.25 to 18.1 kg CO_2_-eq/kg with a reduction of 17.0%, and V50.50 to 14.4 kg CO_2_-eq/kg with a reduction of 33.9% (Tables S11 and S12 in Supplementary Material).

The ingredients, mainly cocoa nibs and cocoa butter, used for the chocolate production, dominate the overall impacts, contributing 94.5–98.2% of EP and 73.5–80.8% of GWP. These results confirm previous findings that cocoa cultivation and processing is the key environmental driver for chocolate [26,100]. Climate impacts of dark chocolate are primarily linked to upstream processes, particularly fertilizer use and direct field emissions during cocoa cultivation, with additional contributions from natural gas use in manufacturing [101].

Using economic allocation, only 1.1% of the cocoa bean’s environmental burden is attributed to CBH, corresponding to 4400 EP/kg and 1.24 kg CO_2_-eq/kg, whereas 98.9% is allocated to nibs. Therefore, substituting nibs with CBH results in substantial reductions in environmental impacts. These reductions would be less pronounced in milk chocolate, where milk accounts for roughly 25% of the climate impact [100].

**Figure 7:**
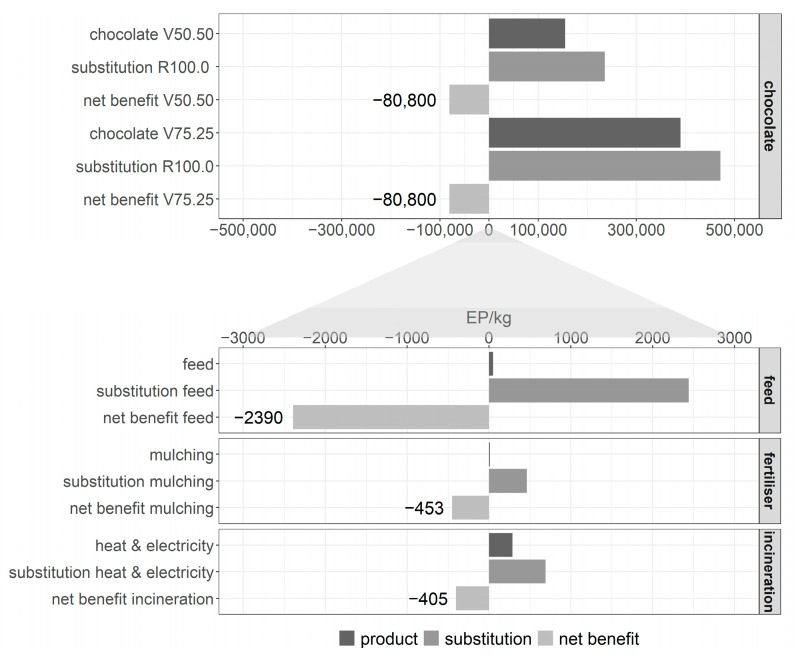
Overall environmental impacts of cocoa bean hull (CBH) valorization and utilization pathways. The assessment was carried out using the ecological scarcity method applying a system expansion approach. The results include the environmental impacts in eco-points (EP) per kilogram of product that incorporates valorized side streams (product), per kilogram of substituted product (substitution) and the net benefit between product and substitution (net benefit). Substitution assumptions: mineral fertilizer with equivalent N–P–K content for mulching; Swiss average electricity mix and natural gas (central/small-scale) for incineration; wheat bran, oat husks, sunflower cake, and alfalfa cubes as nutrient-equivalent feed; recipe R100.0 for chocolate. R100.0 = reference sample, V75.25 = sample with CBH substituting 25% of cocoa nibs, V50.50 = sample with CBH substituting 50% of cocoa nibs.

Beyond chocolate production, CBH can also be valorized as animal feed, soil mulch, or through energy recovery via incineration (Figure 7). A system expansion approach was applied to quantify environmental impacts, substitution effects, and net benefits per kilogram of hulls. Among all pathways, incorporation into chocolate provided the largest environmental gains, with net savings of 88,800 EP (Figure 6) and 21.5 kg CO_2_-eq per kilogram of valorized CBH (Figure S2 in Supplementary Material). In contrast, alternative applications delivered only marginal benefits. Incineration, based on a lower heating value of 19.05 MJ/kg, generated 3.24 MJ/kg of electricity and 6.33 MJ/kg of heat. Substitution of Swiss average electricity and natural gas resulted in net savings of 405 EP/kg or 0.32 kg CO_2_-eq/kg (Tables S13 and S14 in Supplementary Material).

Feed substitution, modelled against a nutritional equivalent mixture of wheat bran, oat husks, sunflower cake, and alfalfa cubes, yielded 2389 EP and 0.28 kg CO_2_-eq per kilogram of CBH. The extent of environmental savings is sensitive to the choice of substituted feed ingredients. For mulching, CBH were modelled as a replacement for mineral fertilizer with equivalent NPK content. This pathway produced only modest benefits (453 EP and 0.26 kg CO_2_-eq), consistent with other studies reporting that fertilizer substitution offers limited environmental advantages [26]. However, additional soil health and carbon sequestration benefits from mulching are likely underrepresented in the applied LCA approaches. The potential to reduce environmental and climate impacts of chocolate by valorizing CBH and integrating them into the chocolate recipe is primarily driven by the reduced demand for cocoa per kilogram of chocolate. In comparison, the benefits derived from using CBH as feed or fertilizer are negligible and, therefore, do not substantially affect the net reduction potential for chocolate (17% for eco-points and 16% for climate impacts).

Overall, the most promising applications of cocoa bean shells lie in food formulations, consistent with previous research [26]. As cocoa is globally limited yet in high demand, increasing pressure on tropical regions has been associated with expanding cultivation to deforestation [102,103]. Improving processing efficiency and valorizing existing biomass streams therefore offer effective pathways to meet demand without additional land conversion. Our findings highlight ingredient substitution as a key lever to reduce the environmental footprint of chocolate production and illustrate the broader potential of side-stream valorization in the food sector. Alternative pathways remain relevant when food applications are restricted by sensory, regulatory, or logistical constraints.

### 3.4. Novel Food Status

According to the EU-Novel-Food-Status-Catalogue (“Novel Food Status Catalogue”, n.d.), the seeds of *Theobroma cacao* L. are not novel, as this product was used for human consumption to a significant degree within the Union before 15 May 1997. This includes the shells surrounding the beans and, therefore, the CBH. As no novel production process has been applied, i.e., a production process which was not used for food production within the EU before 15 May 1997 and which gives rise to significant changes in the composition or structure of the food, affecting its nutritional value, metabolism or level of undesirable substances [104]; there is no change in the novel food status of CBH via production process. Hence, it is an ingredient classified as not novel in food.

## 4. Conclusions

Substituting cocoa nibs with CBH demonstrated functional, nutritional and economical potential but also revealed technological limitations. Increasing CBH content consistently increased viscosity, yield stress, and firmness, likely reflecting the reduced fat phase and high fibre and solids load introduced by the hulls. These effects indicate that processing parameters during milling and conching or emulsifier systems must be further adapted to maintain flowability at higher inclusion levels and allow for optimal moulding. The observed colour shifts and increased firmness underline the need for further optimization to balance sensory acceptance with sustainability gains. Given its non-novel food status and the climate impact reductions (−17% for V75.25 and −34% for V50.50), chocolate represents a viable valorization route for this abundant side stream of cocoa processing. Future work should refine particle size and fat adjustment strategies to enable industrial integration without compromising texture or consumer appeal. In addition, application in non-premium chocolate such as, e.g., chocolate for baking purposes should be considered.

## Figures and Tables

**Table 1:** Composition of the chocolate/cocoa bar productions. R100.0 = reference sample, V75.25 = sample with cocoa bean hulls substituting 25% of cocoa nibs, and V50.50 = sample with cocoa bean hulls substituting 50% of cocoa nibs.

Ingredients [%]	R100.0	V75.25	V50.50
Cocoa nibs	65	48.75	32.5
Cocoa butter	14.5	14.5	14.5
Sugar	20	20	20
Rapeseed lecithin	0.5	0.5	0.5
Cocoa bean hulls (pre-milled)	0	16.25	32.5

**Table 2:** Compositional characterization and properties of cocoa bean hulls.

	Mean	SD
Nutritional facts		
Moisture content [%]	5.9	0.01
Fat [%]	6.4	0.08
Protein [%]	15.5	0.8
Dietary fibre (total) [%]	54.6	7.0
Thereof soluble dietary fibre [%]	14.7	1.9
Thereof insoluble dietary fibre [%]	39.9	6.6
**Critical components**		
Cadmium [mg/kg]	0.37	0.08
Total aflatoxins (B1, B2, G1, G2) [μg/kg]	nd	nd
Ochratoxin A [μg/kg]	0.7	0.3
Acrylamide [μg/kg]	273	55
Aerobic mesophilic germs [CFU/g]	<1000	-
Yeasts [CFU/g]	<100	-
Mould [CFU/g]	<100	-
*Escherichia coli* [CFU/g]	<10	-
*Bacillus cereus* (presumptive) [CFU/g]	<100	-
*Listeria monocytogenes* [CFU/g]	<10	-
**Technofunctional qualities**		
Water-holding capacity [%]	357.2	98.1
Oil-holding capacity [%]	238.1	96.1
Water absorption [% d.m.]	261.4	12.8
pH value [−]	5.3	0.02
Foaming capacity [%]	6.4	1.0
Foaming stability t1 [%]	6.4	1.0
Foaming stability t2 [%]	7.2	1.0
Foaming stability t3 [%]	8.0	1.0
Emulsion stability t1 [%]	93.5	0.2
Emulsion stability t2 [%]	79.6	1.1
Emulsion stability t3 [%]	69.9	3.2
Emulsion stability t4 [%]	57.0	3.3
Emulsion stability t5 [%]	50.5	2.8
x_10,3_ = 10% particle size [µm]	11.8	0.3
x_50,3_ = median particle size [µm]	40.2	2.8
x_90,3_ = 90% particle size [µm]	161.4	5.1

**Table 3:** Rheological parameters following the Windhab model (Ref) with τ0, the yield stress needed to initiate flow, τ1 the extrapolated yield stress at very high shear rate, η∞ the infinite shear viscosity and D* a model parameter related to stress sensitivity. R100.0 = reference sample, V75.25 = sample with cocoa bean hulls substituting 25% of cocoa nibs, V50.50 = sample with cocoa bean hulls substituting 50% of cocoa nibs.

	τ0 (Windhab) [Pa]		τ1 (Windhab) [Pa]		η∞ (Windhab) [mPas]		D* (Windhab) [Pa]	
	Mean	SD	Mean	SD	Mean	SD	Mean	SD
**R100.0**	4.10	0.68	6.57	0.97	525.49	1.80	5.29	0.63
**V75.25**	10.78	0.30	16.34	0.60	1104.37	109.32	5.63	0.12
**V50.50**	37.74	0.38	66.61	1.99	3359.82	36.79	7.08	0.31

**Table 4:** Free fatty acids and hexanal content of chocolates before (t0) and after 3 months of storage (t3m). R100.0 = reference sample, V75.25 = sample with cocoa bean hulls substituting 25% of cocoa nibs, V50.50 = sample with cocoa bean hulls substituting 50% of cocoa nibs.

Sample	Free Fatty Acids [g/100 g]				Hexanal [mg/kg]			
	t0		t3m		t0		t3m	
	Mean	SD	Mean	SD	Mean	SD	Mean	SD
**R100.0**	1.1	0.022	0.92	0.0184	0.3	0.15	0.2	0.1
**V75.25**	1.2	0.024	1.2	0.024	0.2	0.1	0.4	0.2
**V50.50**	1.7	0.034	1.4	0.028	0.3	0.1	0.5	0.25

**Table 5:** Nutritional content of chocolate variants calculated based on the specification sheets of the raw materials (cocoa nibs, cocoa butter, sugar and rapeseed lecithin) and the laboratory measurements for CBH. R100.0 = reference sample, V75.25 = sample with cocoa bean hulls substituting 25% of cocoa nibs, V50.50 = sample with cocoa bean hulls substituting 50% of cocoa nibs.

	R100.0	V75.25	V50.50
**Energy [kcal/100 g]**	605	539	473
**Fat [g/100 g]**	50.4	42.6	34.7
**Thereof saturated fatty** **acids [g/100 g]**	30.7	25.3	20.0
**Carbohydrates [g/100 g]**	23.6	25.6	27.6
**Sugar [g/100 g]**	20.2	20.2	20.1
**Fibres [g/100 g]**	0.0	8.9	17.7
**Protein [g/100 g]**	7.8	8.4	8.9
**Salt [g/100 g]**	0.01	0.01	0.01

## Data Availability

The original contributions presented in this study are included in the article/Supplementary Materials. Further inquiries can be directed to the corresponding author.

## Supplementary Materials

The following supporting information can be downloaded at: https://www.mdpi.com/article/10.3390/foods15030558/s1, Figure S1: Product flow of dried cocoa beans to cocoa bean hulls and toasted cocoa beans; Figure S2: Overall environmental impacts of cocoa bean hull valorization and utilization pathways. The assessment was carried out using the IPCC2021 method applying a system expansion approach. The results include the CO_2_-eq per (1) products that incorporates valorized side streams, (2) substitution products and (3) net benefits resulting from the substitution of corresponding reference products. R100.0 = reference sample, V75.25 = sample with coca bean hulls substituting 25% of cocoa nibs, V50.50 = sample with coca bean hulls substituting 50% of cocoa nibs; Table S1: Outputs to and inputs from technosphere for cocoa mass (nibs) and hulls from 1kg cocoa beans, using economic allocation.; Table S2: Outputs to and inputs from technosphere for cocoa butter, liquor and powder produced from 1 kg cocoa bean, using economic allocation; Table S3: Inventory data for the valorization scenario chocolate. Per kg of dark chocolate; Table S4: physical calorific value per nutrient and the content per kg cocoa hulls of these nutrients; Table S5: Input data for heat and electricity co-creation from incineration from 1 kg cocoa bean hulls and its substitution products; Table S6: Input data for substitution feed mix for 1 kg cocoa bean hulls used as feed; Table S7: input data and its used datasets for the modelling of scenario food and its substitution; Table S8: Phosphate, nitrogen, and potassium content within cocoa bean hulls; Table S9: Input data used for modelling the substitution products (synthetic NPK fertilizer) of BSG- and BR-compost; Table S10: Economic allocation calculation of cocoa bean hulls; Table S11: Results in eco-points of packaging material per production stage (waste, material, moulding), valorization, and allocation; Table S12: Results in CO_2_-eq of kg granola per production stage (ingredients, processing, packaging), valorization, and allocation based on the IPCC2021 GWP100 method; Table S13: Results in eco-points assessed with the ecological scarcity method for all scenarios including substitution products based on system expansion approach per kg of side stream valorized or utilized; Table S14: Results in kg CO_2_-eq of all scenarios including substitution products based on system expansion approach per kg of side stream valorized or utilized.
