# Supplementary material for: Technical Feasibility, Quality and Environmental Impact of a Partial Replacement of Cocoa Nibs with Cocoa Bean Hulls in Chocolate Bars
Source: Foods. 2026 Feb 4;15(3):558. doi: 10.3390/foods15030558 (PMC12897199; doi:10.3390/foods15030558)
Supplement: Supplementary file 1 [file foods-15-00558-s001.zip › foods-4080203-supplementary.pdf]

## Supporting Material - Life Cycle Assessment

For the LCA in this study, detailed inventory data was required for each valorization and utilization scenario of cocoa bean hulls. The following supporting material provides complete inventory data for all scenarios, including explanatory comments and references to the specific datasets used from the ecoinvent database. The second chapter explains the economic allocation approach applied, while the third chapter presents additional results and visualizations.

### 1. Inventory Data

### 1.1. Valorization Scenario - Chocolate

First, the cocoa beans are dried where the hull is separated (Table S1). Table S2 shows the data used for modelling cocoa butter and cocoa liquor.

**Table S1:** *Outputs to and inputs from technosphere for cocoa mass (nibs) and hulls from 1kg cocoa beans, using economic allocation.*

| Outputs to technosphere | Amount | Unit | comment/source |
|---|---|---|---|
| VALISS Cocoa mass (nibs) | 0.83 | Kg | 98.9% allocation (see Table S10) |
| VALISS Cocoa hull | 0.17 | Kg | 1.1% allocation (see Table S10) |

| Inputs form technosphere | Amount | Unit | comment/source |
|---|---|---|---|
| Cocoa bean {GLO}\| market for cocoa bean | 1 | kg | (Ecoinvent, 2025) |

**Table S2:** *Outputs to and inputs from technosphere for cocoa butter, liquor and powder produced from 1 kg cocoa bean, using economic allocation.*

| Outputs to technosphere | Amount | Unit | comment/source |
|---|---|---|---|
| Cocoa butter/RER | 0.23125 | Kg | 51.3% allocation (see Table S10) |
| Cocoa liquor/RER | 0.31948 | Kg | 42.1% allocation (see Table S10) |
| Cocoa powder/RER | 0.075 | kg | 6.6% allocation (see Table S10) |

| Inputs form technosphere | Amount | Unit | comment/source |
|---|---|---|---|
| Electricity, low voltage {ENTSO-E}\| market group for electricity, low voltage | 0.0881 | kWh | (Ecoinvent, 2025) |
| Transport, freight, lorry, diesel, unspecified {RER}\| market for transport, freight, lorry, unspecified | 1.4916 | tkm | (Ecoinvent, 2025) |
| Transport, passenger, car, fleet average {RER}\| market for transport, passenger, car, fleet average | 0.23911 | km | (Ecoinvent, 2025) |
| Tap water {Europe without Switzerland}\| market for tap water | 5.1274 | Kg | (Ecoinvent, 2025) |
| Cocoa bean {GLO}\| market for cocoa bean | 1 | kg | (Ecoinvent, 2025) |

Table S3 provides an overview of all input data used for chocolate, including their calculations and quantities.

**Table S3:** *Inventory data for the valorization scenario chocolate. Per kg of dark chocolate. All datasets are from the background database ecoinvent (Ecoinvent, 2025).*

| | source | dataset & comment | Unit | R | 16.25% hulls | 23.5% hulls |
|---|---|---|---|---|---|---|
| Ingredients | See Table S1 | VALISS Cocoa mass (nibs) | g | 650 | 487 | 325 |
| | See Table S2 | Cocoa butter/RER | g | 145 | 145 | 145 |
| | VALISS | Sugar, from sugar beet {CH}\| sugar beet processing | g | 200 | 200 | 200 |
| | VALISS | Soybean lecithin (solvent), at processing {DE} Economic, U | g | 5 | 5 | 5 |
| | VALISS | VALISS Cocoa hull | g | 0 | 163 | 325 |
| processing | Energy consumed for chocolate production (Halba, 2013) | Electricity, low voltage {CH}\| market for electricity, low voltage | kwh | 0.950 | 0.950 | 0.950 |
| | Water used for production (Halba, 2013) | Tap water {CH}\| market for tap water | kg | 1.75 | 1.75 | 1.75 |
| | Heat used in production (Halba, 2013) | • Heat, district or industrial, other than natural gas {CH}\| heat production, light fuel oil, at industrial furnace 1MW<br>• Heat, district or industrial, natural gas {Europe without Switzerland}\| heat production, natural gas, at industrial furnace >100kW<br>• Heat, district or industrial, other than natural gas {CH}\| heat, from municipal waste incineration to generic market for heat, district or industrial, other than natural gas | MJ | 0.456 | 0.456 | 0.456 |
| valorization | Milling machine (Retsch, 2025) | Industrial machine, heavy, unspecified {RER}\| market for industrial machine, heavy, unspecified<br><br>assumptions – milling with sr300 retsch impact mill<br>- machine type: impact mill (sr300, retsch)<br>- assumed lifespan: 15 years<br>- machine weight: 60 kg<br><br>processing:<br>- batch volume: 5 l<br>- density of material: 0.1–0.15 g/cm³<br>- actual batch mass: approx. 250 g (cannot be filled completely)<br>- time per batch: 23.4 minutes<br>→ processing rate: 641 g/hour<br><br>annual and lifetime throughput:<br>operating time:<br>- 10 hours/day, 5 days/week, 52 weeks/year → 2,600 hours/year<br>- annual throughput: 0.641 kg/h×2,600 h=1,666.6 kg/year<br>- total lifetime throughput (15 years): 1,666.6 kg/year×15=24,999 kg<br>- value: 60/24999 = 0.0024 kg per kg of cocoa milling<br>  o 0.0024/1000*162.5 = 0.00039<br>  o 0.0024/1000*325 = 0.00078 | kg | 0 | 0.00039 | 0.00078 |
| | Electricity for milling (Retsch, 2025) | Electricity, low voltage {CH}\| market for electricity, low voltage<br><br>milling of cocoa hulls to 500 microns:<br>- energy consumption: 0.539 kwh per 777 g (primary data)<br>- target amount: 162.5 or 325g cocoa hulls<br>- calculated energy use:<br><br>0.539 kwh/ 777 g *162.5g = 0.1127 kwh<br>0.539 kwh/ 777 g *325g = 0.2255 kwh | kwh | 0 | 0.113 | 0.226 |

| | | | | | | |
|---|---|---|---|---|---|---|
| **packaging & sale** | (Halba, 2013) | Folding boxboard carton {RER}\| folding boxboard carton production | kg | 0.0160 | 0.0160 | 0.0160 |
| | | Corrugated board box {RER}\| corrugated board box production | kg | 0.195 | 0.195 | 0.195 |
| | | Kraft paper {RER}\| kraft paper production | kg | 0.0205 | 0.0205 | 0.0205 |
| | | Packaging film, low density polyethylene {RER}\| packaging film production, low density polyethylene | kg | 0.0070 | 0.0070 | 0.0070 |
| | | Aluminium, primary, ingot {RoW}\| market for aluminium, primary, ingot | kg | 0.0070 | 0.0070 | 0.0070 |
| | | Sheet rolling, aluminium {RER}\| sheet rolling, aluminium | kg | 0.0070 | 0.0070 | 0.0070 |
| | | Carton board box production, with offset printing {CH}\| carton board box production service, with offset printing | kg | 0.0365 | 0.0365 | 0.0365 |
| | | Building, hall {CH}\| building construction, hall | M2 | 0.0001 | 0.0001 | 0.0001 |
| | | Transport, freight, sea, transoceanic ship {GLO}\| market for transport, freight, sea, transoceanic ship | Tkm | 4.86 | 4.86 | 4.86 |
| | | Transport, freight, train, fleet average {Europe without Switzerland}\| market for transport, freight, train, fleet average | Tkm | 0.454 | 0.454 | 0.454 |
| | | Transport, freight, lorry, diesel, unspecified {GLO}\| market group for transport, freight, lorry, diesel, unspecified | tkm | 0.125 | 0.125 | 0.125 |

## 1.2 Utilization Scenario – incineration of cocoa bean hulls

The scenario incineration is based on the approach of Beretta et al. (2017). For the life cycle inventory of the incineration, an existing eco-inventory from the Ecoinvent database for the incineration of biowaste was used for all by-streams: *Biowaste {GLO}| treatment of biowaste, municipal incineration* . During incineration, heat and electricity are generated. The amount of produced electricity and heat was determined proportionally to the lower heating value.

The heating value was calculated considering the nutrient composition using following equation:

$$H_u = P * B_P + F * B_F + C * B_C + Fi * B_{Fi} - W * B_W$$

With P = protein content, F = fat content, C = carbohydrate content, $F_i$ = fiber content, W = water content, $B_i$ = physical calorific value of the nutrient

**Table S4:** *physical calorific value per nutrient and the content per kg cocoa hulls of these nutrients. Cocoa hulls nutrition is obtained as primary data within this study.*

| Nutrition | physical calorific value of the nutrient | Cocoa hulls nutrition per kg |
|---|---|---|
| P = protein content | 23 MJ/kg | 0.155kg (VALISS) |
| F = fat content | 38.9 MJ/kg | 0.064kg (VALISS) |
| C = carbohydrate content | 17.2 MJ/kg | 0.176kg (VALISS) |
| $F_i$ = fiber content | 18 MJ/kg | 0.546kg (VALISS) |
| W = water content | -2.441 MJ/kg | 0.059kg (VALISS) |

Based on the values in Table S4, the equation can be calculated:

$$H_u = 0.155kg * 23\frac{\mathrm{MJ}}{\mathrm{kg}} + 0.064kg * 38.9\frac{\mathrm{MJ}}{\mathrm{kg}} + 0.176kg * 17.2\frac{\mathrm{MJ}}{\mathrm{kg}} + 0.546kg * 18\frac{\mathrm{MJ}}{\mathrm{kg}} - 0.059kg * -2.441\frac{\mathrm{MJ}}{\mathrm{kg}}$$

$$H_u = 19.05\ MJ/kg$$

The 19.05 MJ/kg lower heating value does align with results from Salcedo-Puerto et al. (2025) who concluded a lower heating value between 17 and 23 MJ/kg of cocoa residues.

The resulting amount of electricity and heat from incineration was calculated using the current Swiss average efficiency of electricity and heat recovery from municipal solid waste incineration plants. The electricity efficiency amounts to 17%, and the heat efficiency to 33.2% (Bendig et al., 2024). The following are the calculated heating values as well as the resulting electricity and heat production from the incineration of cocoa hulls:

- lower heat value: 19.05 MJ/kg fresh mass
- the electricity production is 3.24 MJ/kg fresh mass
- heat production 6.33 MJ/kg fresh mass

The life cycle inventories of the substitutable products were approximated using existing datasets from Ecoinvent. It was assumed that the produced electricity substitutes the Swiss consumer mix, while the produced heat substitutes heat from natural gas. All used input data and datasets are shown in Table S5.

**Table S5:** *Input data for heat and electricity co-creation from incineration from 1kg cocoa bean hulls and its substitution products.*

| scenario | FU | input | dataset/source | amount | unit | comment |
|---|---|---|---|---|---|---|
| Incineration – heat | 6.33 MJ | Cocoa bean hulls | VALISS Cocoa bean hulls (see Table S1) | 1 | kg | 1kg cocoa bean hulls --> system expansion and therefore 0 |
| | | transport | Transport, freight, lorry, diesel, unspecified {RER}\| market for transport, freight, lorry, unspecified (Ecoinvent, 2025) | 0.01 | tkm | assumed distance for side stream transportation of 10 km |
| | | incineration | Biowaste {GLO}\| treatment of biowaste, municipal incineration (Ecoinvent, 2025) | 1 | kg | incineration of the 1kg cocoa bean hulls |
| Incineration - electricity | 3.42 MJ | Cocoa bean hulls | VALISS Cocoa bean hulls (see Table S1) | 1 | kg | 1kg cocoa bean hulls --> system expansion and therefore 0 |
| | | transport | Transport, freight, lorry, diesel, unspecified {RER}\| market for transport, freight, lorry, unspecified (Ecoinvent, 2025) | 0.01 | tkm | assumed distance for side stream transportation of 10 km |
| | | incineration | Biowaste {GLO}\| treatment of biowaste, municipal incineration (Ecoinvent, 2025) | 1 | kg | incineration of the 1kg cocoa bean hulls |
| Heat substitution | 6.33 MJ | Heat swiss | Heat, central or small-scale, natural gas {CH}\| market for heat, central or small-scale, natural gas (Ecoinvent, 2025) | 6.33 | MJ | |
| Electricity substitution | 3.42 MJ | Swiss electricity mix | Electricity, medium voltage {CH}\| market for electricity, medium voltage (Ecoinvent, 2025) | 3.42 | MJ | |

### 1.3 Utilization Scenario – feed of cocoa bean hulls

For the modelling of the scenario feed, cocoa bean hulls are used as feed for animals. For this a 10km transport distance of the side stream (as assumed to be locally used) is assumed.

As a substitution for the 1kg cocoa bean hulls feed, a feed composition with similar nutritional value is used. Cocoa bean hulls as a single feed material are relatively variable in their contents. Their nutrient composition is not particularly consistent, as it depends strongly on factors such as the origin of the beans, processing, the proportion of residual pulp or foreign matter, and the degree of drying. Cocoa hulls contain theobromine. In equestrian sports, theobromine is considered doping, even in trace amounts. For this reason, UFA completely avoids the use of cocoa shells in feed formulations. Nevertheless, a possible "substitute feed mix" for one ton of cocoa shells (Table S6) has been suggested by Jacques Emmenegger, the head of technical services, who is also responsible for the feed formulations (UFA, 2025).

**Table S6:** *Input data for substitution feed mix for 1 kg cocoa bean hulls used as feed (UFA, 2025).*

| input | dataset and source | amount | unit |
|---|---|---|---|
| wheat bran | Wheat bran, IP, at industrial mill/CH U (ZHAW, 2025) | 600 | g |
| oat husks | Oat husk meal, at processing {BE} Economic, U (ZHAW, 2025) | 150 | g |
| sunflower cake | Sunflower meal IP, at oil mill/CH U (Ecoinvent, 2025) | 150 | g |
| alfalfa cubes 16% | Alfalfa-grass mixture, Swiss integrated production {CH}\| alfalfa-grass mixture production, Swiss integrated production (Ecoinvent, 2025) | 100 | g |

The feed of cocoa bean hulls include the transportation of the materials to the pellet production location, the pellet production and then the transportation of the pellets from the production site to the retail. The data on electricity consumption for the pellet production and the heat consumption for the drying is taken from a study on greenhouse gas emissions of feed production and utilization (Vellinga et al., 2013). The same processes are used for the substitution product but with other input materials as listed in Table S6. All input data and datasets used are shown in Table S7.

**Table S7:** *input data and its used datasets for the modelling of scenario food and its substitution.*

| scenario | FU | input | dataset | amount | unit | comment/source |
|---|---|---|---|---|---|---|
| Feed cocoa bean hulls | 1kg | Feed material | cocoa bean hulls - VALISS {CH} (see Table S1) | 1 | kg | 0kg as system expansion |
| | | Transport of side stream | Transport, freight, lorry, diesel, unspecified {RER}\| market for transport, freight, lorry, unspecified (Ecoinvent, 2025) | 0.01 | tkm | assumed distance for side stream transportation of 10 km |
| | | Processing electricity – pressing in pellets | Electricity, low voltage {CH}\| market for electricity, low voltage (Ecoinvent, 2025) | 0.315 | MJ | Vellinga, T. V., Blonk, H., Marinussen, M., Zeist, W. J. Van, |

| | | | | | | |
|---|---|---|---|---|---|---|
| | | Processing heat - drying | Heat, central or small-scale, natural gas {CH}\| market for heat, central or small-scale, natural gas (Ecoinvent, 2025) | 0.135 | MJ | Boer, I. J. M. De, & Starmans, D. (2013). Methodology used in feedprint: a tool quantifying greenhouse gas emissions of feed production and utilization. |
| | | Transport from feed production to animals | Transport, freight, lorry, diesel, unspecified {RER}\| market for transport, freight, lorry, unspecified (Ecoinvent, 2025) | 0.07 | tkm | assumed distance for side stream transportation of 70 km |
| Feed substitution | 1kg | Feed material | See Table S6 | | | |
| | | Transport of side stream | Transport, freight, lorry, diesel, unspecified {RER}\| market for transport, freight, lorry, unspecified (Ecoinvent, 2025) | 0.01 | tkm | assumed distance for side stream transportation of 10 km |
| | | Processing electricity – pressing in pellets | Electricity, low voltage {CH}\| market for electricity, low voltage (Ecoinvent, 2025) | 0.315 | MJ | Vellinga, T. V., Blonk, H., Marinussen, M., Zeist, W. J. Van, Boer, I. J. M. De, & Starmans, D. (2013). |
| | | Processing heat - drying | Heat, central or small-scale, natural gas {CH}\| market for heat, central or small-scale, natural gas (Ecoinvent, 2025) | 0.135 | MJ | |
| | | Transport from feed production to animals | Transport, freight, lorry, diesel, unspecified {RER}\| market for transport, freight, lorry, unspecified (Ecoinvent, 2025) | 0.07 | tkm | assumed distance for side stream transportation of 70 km |

### 1.4 Utilization Scenario – mulching with cocoa bean hulls

The use of cocoa bean hulls as an organic mulch or soil amendment is widespread (Rojo-Poveda et al., 2020). Therefore, cocoa bean hulls are modelled as mulching.

The first drawback of using cocoa bean shells (CBS) as mulch is their low bulk density. Being very lightweight, they can alter soil structure and negatively affect soil properties when applied in large quantities (Rojo-Poveda et al., 2020).

The second drawback of cocoa shell mulch is its unfavorable C/N ratio (typically 30–40, and in some cases above 50). This imbalance leads to nitrogen immobilization during decomposition, reducing the immediate availability of nitrogen to plants (Lykas et al., 2019). In practice, this often necessitates the application of additional mineral fertilizers to offset the temporary nitrogen deficit (Kaba et al., 2019). A similar issue arises with composted cocoa shells: while mature composts are generally expected to have a C/N ratio below 25 (Sangamithirai et al., 2015), cocoa shell composts frequently exceed this threshold, which can further exacerbate nitrogen deficiency, especially in soils already low in nitrogen (Lykas et al., 2019).

However, nitrogen, phosphorus, and potassium content can be a potential benefit for improving soil quality when applied as mulch or fertilizer (Rojo-Poveda et al., 2020). The mineralization of organic nitrogen in cocoa shells into plant-available nitrate and ammonium is a slow process that can take several months. This delayed release has advantages, as it provides nutrients over an extended period, but also drawbacks, since the supply may not coincide with the plants' peak nutrient demand.

Therefore, mulching was analyzed as one option of utilizing cocoa bean hulls.

The nutrients within cocoa shells is not well researched. P content is around 0.5-1g/100g and K content between 1.25 and 1.82g/100g (Rojo-Poveda et al., 2020). Assuming both, mulching is applied, they are substituted with the mineral fertilizer amount saved through that application (Table S8). For the modelling of the scenario mulching with cocoa bean hulls it either replaces mulching with another residue of plants or it replaces fertilizing. In this study, fertilizing is assumed.

**Table S8**: *Phosphate, nitrogen, and potassium content within cocoa bean hulls. Nutrient content is based on values from Rojo-Poveda et al. (2020).*

| **mulching (used as hulls)** | **fertilizer** |
|---|---|
| 2.5% of DM is Nitrogen → 25g | N - $NH_4^+$ and $NO_3^-$ |
| 1.33–2.29% of DM is Phosphate →13.3-22.9g | P – $P_2O_5$ |
| 1.50–2.19% of DM is K2O → 15-21.9g | K – $K_2O$ |

The life cycle inventories of the substitutable products were approximated using existing inventories from the Ecoinvent database (Table S9). The mulching is approximated with inventories of mineral N, $P_2O_5$, and $K_2O$ fertilizers, respectively.

**Table S9:** *Input data used for modelling the substitution products (synthetic NPK fertilizer) of BSG- and BR-compost. N,P, and K input is based on values from Rojo-Poveda et al. (2020).*

| input | Amount | unit | dataset/source |
|---|---|---|---|
| P-fertilizer | 0.02 | kg | Inorganic phosphorus fertiliser, as P2O5 {CH}\| market for inorganic phosphorus fertiliser, as P2O5 (Ecoinvent, 2025) |
| K-fertilizer | 0.018 | kg | Inorganic potassium fertiliser, as K2O {CH}\| market for inorganic potassium fertiliser, as K2O (Ecoinvent, 2025) |
| N-fertilizer | 0.025 | kg | Inorganic nitrogen fertiliser, as N {CH}\| market for inorganic nitrogen fertiliser, as N (Ecoinvent, 2025) |

## 2. Economic Allocation

The economic allocation was carried out for cocoa bean hulls. All calculations are in Table S10. An overview of the sidestreams is provided in Figure S1. For cocoa nibs the allocation factor is 98.9% and for cocoa hulls 1.1%.

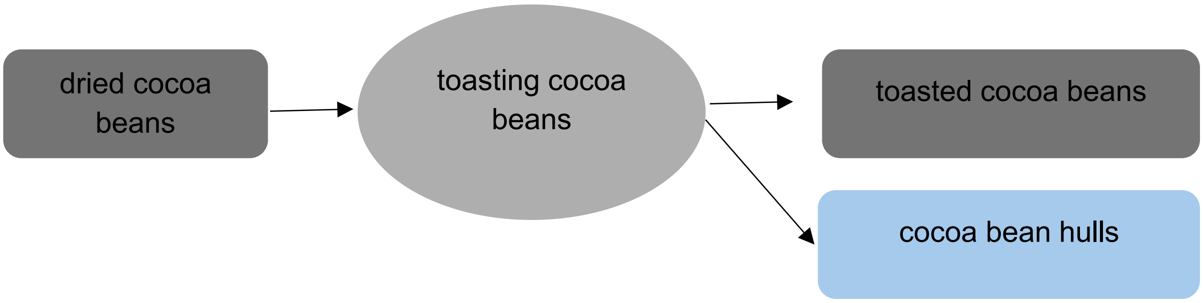


**Figure S1:** *Product flow of dried cocoa beans to cocoa bean hulls and toasted cocoa beans*

**Table S10:** *Economic allocation calculation of cocoa bean hulls.*

| **product** | | **economic value** | **source** |
|---|---|---|---|
| Cocoa nibs | 0.83 kg per kg dried cocoa beans | 15 CHF/ kg | prices between 15 and CHF/kg. Assumtion B2B 10-18 CHF/kg (Trading Economics, 2025) |
| Cocoa hulls | 0.17 kg per kg dried cocoa beans | confidential | primary data from project |
| **value added** | | | |
| Cocoa nibs | 12.45 CHF | | |
| Cocoa hulls | confidential | | |
| | | | |
| **allocation factor** | | | |
| Cocoa nibs | 0.989 | | |
| Cocoa hulls | 0.0101 | | |

## 3. Results

### 3.1. Results of chocolate with economic allocation

The results in eco-points (Table S11) and $CO_2$-eq (Table S12) for the more detailed production stages of chocolate production, including valorisation and allocation, are presented below.

**Table S11:** *Results in eco-points of packaging material per production stage (waste, material, moulding), valorization, and allocation.*

| scenario | production | | | valorisation | allocation | sum |
|---|---|---|---|---|---|---|
| | ingredients | processing | retail | | | |
| R100.0 | 75100 | 295 | 1090 | 0 | 0 | 76500 |
| V75.25 | 62000 | 295 | 1090 | 24 | 715 | 63300 |
| V50.50 | 48800 | 295 | 1090 | 48 | 1430 | 50200 |

**Table S12:** *Results in $CO_2$-eq of kg granola per production stage (ingredients, processing, packaging), valorization, and allocation based on the IPCC2021 GWP100 method.*

| scenario | production | | | valorisation | allocation | sum |
|---|---|---|---|---|---|---|
| | ingredients | processing | retail | | | |
| R100.0 | 2.67 | 0.117 | 0.520 | 0.000 | 0.000 | 3.31 |
| V75.25 | 2.23 | 0.117 | 0.520 | 0.004 | 0.024 | 2.87 |
| V50.50 | 1.80 | 0.117 | 0.520 | 0.009 | 0.048 | 2.43 |

## 3.2. Results of all scenarios with avoided burden, system expansion

Table S13 shows the absolute eco-points and table S14 the $CO_2$-eq of the utilized scenarios, their reference products which are substituted and the resulting net benefit.

**Table S13:** *Results in eco-points assessed with the ecological scarcity method for all scenarios including substitution products based on system expansion approach per kg of side stream valorized or utilized.*

| scenario | amount | utilized product | amount | reference product | net benefit |
|---|---|---|---|---|---|
| incineration heat and electricity co-creation of cocoa bean hulls | 9.57 MJ | 286 | 6.33 MJ Heat 3.24 MJ Electr. | 691 | -405 |
| Mulching/ Fertilizer (NPK) | 1kg | 10 | 0.063kg | 462 | -453 |
| feed | 1kg | 49 | 1kg | 2438 | -2389 |
| chocolate v75.25 | 6.15kg | 410118 | 6.15kg | 489691 | -81000 |
| chocolate v50.50 | 3.08kg | 165541 | 3.08kg | 245244 | -81000 |

**Table S14:** *Results in kg $CO_2$-eq of all scenarios including substitution products based on system expansion approach per kg of side stream valorized or utilized.*

| scenario | amount | utilized product | amount | reference product | net benefit |
|---|---|---|---|---|---|
| incineration heat and electricity co-creation of cocoa bean hulls | 9.57 MJ | 0.13 | 6.33 MJ Heat 3.24 MJ Electr. | 0.46 | -0.32 |
| mulching | 1kg | 0.01 | 0.063kg | 0.27 | -0.26 |
| feed | 1kg | 0.02 | 1kg | 0.3 | -0.28 |
| chocolate v75.25 | 6.15kg | 113 | 6.15kg | 134 | -21.5 |
| chocolate v50.50 | 3.08kg | 45.6 | 3.08kg | 67.2 | -21.5 |

## 3.3. Greenhouse gas emissions of all scenarios with avoided burden, system expansion

Figure S2 shows the net benefits resulting from the different scenarios. As described in the main publication, food valorization pathways are saving the most environmental benefit. Using CBH for chocolate results in a net benefit of 22 kg $CO_2$-eq/kg valorized CBH, while all other utilization scenarios show net savings below 0.5 kg $CO_2$-eq/kg valorized CBH.

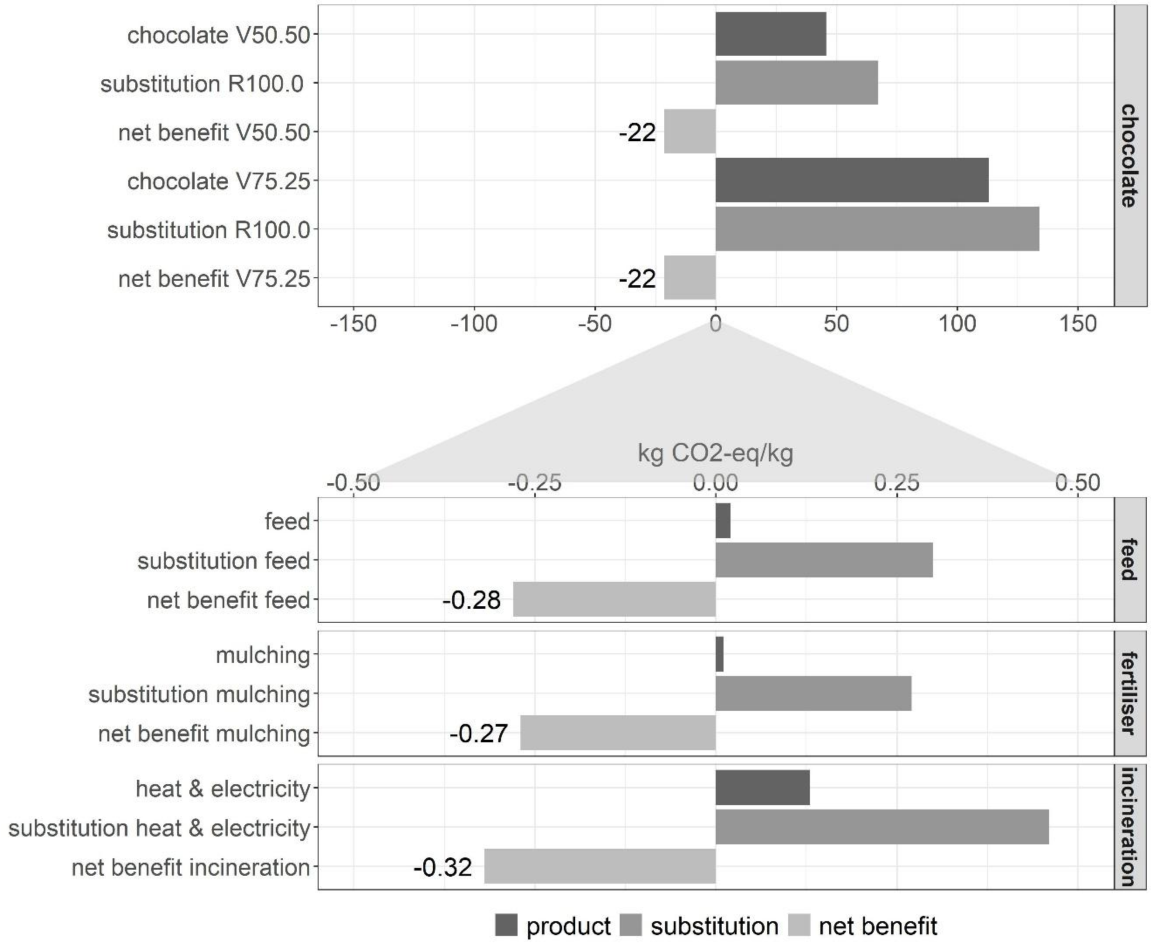


**Figure S2:** *Overall environmental impacts of cocoa bean hull valorization and utilization pathways. The assessment was carried out using the IPCC2021 method applying a system expansion approach. The results include the CO2-eq per 1) products that incorporates valorized side streams, 2) substitution products and, 3) net benefits resulting from the substitution of corresponding reference products. R100.0 = reference sample, V75.25 = sample with coca bean hulls substituting 25% of cocoa nibs, V50.50 = sample with coca bean hulls substituting 50% of cocoa nibs.*

## 4. Literature

1) Chocolats Halba. (2013). Mehrwert durch Nachhaltigkeit. Nachhaltigkeitsbericht 2012

2) UFA AG (2025): Futtermittel-Ersatz für Kakaoschalen. Personal communication with Jacques Emmenegger, 17.06.25.

3) Bendig, M., Hügi, M., Quartier, R., Chrenko, R., & Kiener, M. (2024). *KVA-Bericht 2023 veröffentlicht • Rytec AG*. Bundesamt für Energie (BFE). https://rytec.ch/kva-bericht-2023-veroeffentlicht-dummy/

4) Beretta, C., Stucki, M., & Hellweg, S. (2017). Environmental Impacts and Hotspots of Food Losses: Value Chain Analysis of Swiss Food Consumption. *Environmental Science & Technology*, *51*(19), 11165–11173. https://doi.org/10.1021/acs.est.6b06179

5) Ecoinvent. (2025). Ecoinvent v3.11. *Ecoinvent*. https://ecoinvent.org/ecoinvent-v3-11/

6) Kaba, J. S., Zerbe, S., Agnolucci, M., Scandellari, F., Abunyewa, A. A., Giovannetti, M., & Tagliavini, M. (2019). Atmospheric nitrogen fixation by gliricidia trees (Gliricidia sepium (Jacq.) Kunth ex Walp.) intercropped with cocoa (Theobroma cacao L.). *Plant and Soil*, *435*(1), 323–336. https://doi.org/10.1007/s11104-018-3897-x

7) Lykas, C., Gougoulias, N., & Vagelas, I. (2019). Effect of Manure and Cocoa Shell Biomass Addition on Soil Chemical Properties Under Laboratory Incubation Conditions. *International Journal of Agriculture & Environmental Science*, *6*(6), 58–66. https://doi.org/10.14445/23942568/IJAES-V6I6P109

8) Retsch. (2025). *Rotor Beater Mill SR 300—RETSCH*. Retsch Milling & Sieving. https://www.retsch.com/products/milling/rotor-mills/sr-300/

9) Rojo-Poveda, O., Barbosa-Pereira, L., Zeppa, G., & Stévigny, C. (2020). Cocoa Bean Shell—A By-Product with Nutritional Properties and Biofunctional Potential. *Nutrients*, *12*(4), 1123. https://doi.org/10.3390/nu12041123

10) Salcedo-Puerto, O., Mendoza-Martinez, C., & Vakkilainen, E. (2025). Solid residues from cocoa production chain: Assessment of thermochemical valorization routes. *Renewable and Sustainable Energy Reviews*, *208*, 115048. https://doi.org/10.1016/j.rser.2024.115048

11) Sangamithirai, K. M., Jayapriya, J., Hema, J., & Manoj, R. (2015). Evaluation of in-vessel co-composting of yard waste and development of kinetic models for co-composting. *International Journal of Recycling of Organic Waste in Agriculture*, *4*(3). https://doi.org/10.1007/s40093-015-0095-1

12) Trading Economics. (2025). *Cocoa Price Chart. Historical Data.* Trading Economics - Cocoa. https://tradingeconomics.com/commodity/cocoa

13) Vellinga, T. V., Blonk, H., Marinussen, M., Zeist, W. J. van, & Starmans, D. a. J. (2013). *Methodology used in FeedPrint: A tool quantifying greenhouse gas emissions of feed production and utilization*. https://research.wur.nl/en/publications/methodology-used-in-feedprint-a-tool-quantifying-greenhouse-gas-e

14) ZHAW. (2025). *Agri-food Database, www.zhaw.ch/IUNR/agri-food*. ZHAW Institute for Natural Resource Sciences. https://www.zhaw.ch/de/forschung/projekt/71134

## 5. List of Figures

Figure S1: Product flow of dried cocoa beans to cocoa bean hulls and toasted cocoa beans ....10
Figure S2: Overall environmental impacts of cocoa bean hull valorization and utilization pathways. The assessment was carried out using the IPCC2021 method applying a system expansion approach. The results include the CO2-eq per 1) products that incorporates valorized side streams, 2) substitution products and, 3) net benefits resulting from the substitution of corresponding reference products. R100.0 = reference sample, V75.25 = sample with coca bean hulls substituting 25% of cocoa nibs, V50.50 = sample with coca bean hulls substituting 50% of cocoa nibs. ....13

## 6. List of Tables

Table S1: Outputs to and inputs from technosphere for cocoa mass (nibs) and hulls from 1kg cocoa beans, using economic allocation.... 1
Table S2: Outputs to and inputs from technosphere for cocoa butter, liquor and powder produced from 1 kg cocoa bean, using economic allocation. .... 1
Table S3: Inventory data for the valorization scenario chocolate. Per kg of dark chocolate.... 2
Table S4: physical calorific value per nutrient and the content per kg cocoa hulls of these nutrients. .... 4
Table S5: Input data for heat and electricity co-creation from incineration from 1kg cocoa bean hulls and its substitution products. .... 5
Table S6: Input data for substitution feed mix for 1 kg cocoa bean hulls used as feed. .... 6
Table S7: input data and its used datasets for the modelling of scenario food and its substitution. .... 6
Table S8: Phosphate, nitrogen, and potassium content within cocoa bean hulls. .... 8
Table S9: Input data used for modelling the substitution products (synthetic NPK fertilizer) of BSG- and BR-compost. .... 9
Table S10: Economic allocation calculation of cocoa bean hulls. ....10
Table S11: Results in eco-points of packaging material per production stage (waste, material, moulding), valorization, and allocation. ....11
Table S12: Results in $CO_2$-eq of kg granola per production stage (ingredients, processing, packaging), valorization, and allocation based on the IPCC2021 GWP100 method. ....11
Table S13: Results in eco-points assessed with the ecological scarcity method for all scenarios including substitution products based on system expansion approach per kg of side stream valorized or utilized. ....12
Table S14: Results in kg $CO_2$-eq of all scenarios including substitution products based on system expansion approach per kg of side stream valorized or utilized. ....12
